# Draft Genome Sequence of *Acidihalobacter ferrooxidans* DSM 14175 (Strain V8), a New Iron- and Sulfur-Oxidizing, Halotolerant, Acidophilic Species

**DOI:** 10.1128/genomeA.00413-17

**Published:** 2017-05-25

**Authors:** Himel N. Khaleque, Joshua P. Ramsay, Riley J. T. Murphy, Anna H. Kaksonen, Naomi J. Boxall, Elizabeth L. J. Watkin

**Affiliations:** a CHIRI Biosciences, School of Biomedical Sciences, Curtin University, Perth, Australia; b CSIRO Land and Water, Perth, Australia

## Abstract

The use of halotolerant acidophiles for bioleaching provides a biotechnical approach for the extraction of metals from regions where high salinity exists in the ores and source water. Here, we describe the first draft genome of a new species of a halotolerant and iron- and sulfur-oxidizing acidophile, *Acidihalobacter ferrooxidans* DSM-14175 (strain V8).

## GENOME ANNOUNCEMENT

The halotolerant acidophile *Acidihalobacter prosperus* is well known for its ability to oxidize iron at low pH under saline conditions (1, 2). *A. ferrooxidans* DSM 14175 (strain V8) represents a similar group of Gram-negative, mesophilic, halotolerant acidophiles that also has the ability to oxidize iron and sulfur and has a chemolithoautotrophic lifestyle. It was isolated from the same shallow acidic pool at the Aeolian Islands of Italy as *A. prosperus* DSM 14174 (strain V6) (3) and was found to dominate mixed cultures during mesophilic pyrite oxidation (4).

Total DNA was extracted from *A. ferrooxidans* DSM 14175 using the modified method of nucleic acid extraction for acidophiles, as described by Zammit et al. (5). DNA was sequenced using Illumina MiSeq (619,160 paired-end reads, 2 × 300-bp reads) and PacBio RS SMRT sequencing technologies (733,419 subreads with a mean read length of 1,602 bp). *De novo* hybrid assembly using SPAdes version 3.9.0 (6) generated 10 contigs, which were then used with PacBio reads to generate a scaffold using SSPACE-LongRead version 1.1 (7). The resulting scaffold was a single circular chromosome with an approximate size of 3,448,835 bp (with 4 gaps with a total approximate size of 6 kb) with approximately 13× Illumina read depth and 355× PacBio read depth. The genes were annotated using the NCBI Prokaryotic Genome Annotation Pipeline version 3.3 and GeneMarkS+. The genome has a G+C content of 61.6% and contains 45 tRNA sequences, 1 rRNA operon, and 3,089 protein-coding genes.

Similar to the genomes of *A. prosperus* DSM 5130 and DSM 14174, genome analysis of *A. ferrooxidans* DSM 14175 showed the presence of the *rus* operon genes for iron oxidation (8–10).

Also found were genes for carboxysomes and carbon dioxide fixation through the Calvin-Benson-Bassham cycle (9–11) and those for nitrogen fixation through the Nif complex (9, 10, 12). A complete set of genes for chemotaxis and flagellar biosynthesis, similar to those found in *A. prosperus* strains DSM 5130 and DSM 14174, were also present (9, 10). However, unlike the genomes of the *A. prosperus* strains, the genome of *A. ferrooxidans* DSM 14175 does not contain genes encoding the SoxAX, B, and YZ subunits of the sulfur oxidation system (9, 10, 13); rather, it contains genes encoding sulfur oxygenase reductases, which may be responsible for sulfur metabolism in this strain (13).

The genome of *A. ferrooxidans* DSM 14175 has genes for pathways involved in tolerance to stresses such as acid and oxidative stress. Considering the ability of this strain to withstand high osmotic stress in a low-pH environment, some of the most important stress-tolerance genes are those encoding operons for the biosynthesis and regulation of ectoine, glycine betaine, and osmoregulated periplasmic glucan, as well as for glycine betaine and choline uptake (14, 15). These proteins act as compatible solutes in acidophiles under osmotic stress and may provide assistance in the survival of halotolerant acidophiles (14, 15).

### Accession number(s).

The whole genome of *A. ferrooxidans* DSM 14175 (strain V8) has been deposited at DDBJ/EMBL/GenBank under the accession number CP019434. The version described in this paper is the first version, CP019434.1.
